# Bone conducted cervical vestibular evoked myogenic potentials: comparison of tone burst stimulus vs chirp stimulus

**DOI:** 10.1007/s00405-024-08886-5

**Published:** 2024-08-04

**Authors:** Ceren Karaçaylı, Ercan Karababa, Bülent Satar

**Affiliations:** 1 Department of Otorhinolaryngology, University of Health Sciences, Gülhane Traning and Research Hospital, Ankara, Turkey; 2 Department of Audiology, Gulhane Faculty of Health Sciences, University of Health Sciences, Ankara, Turkey; 3 Department of Otorhinolaryngology, University of Health Sciences Gulhane Faculty of Medicine, Ankara, Turkey

**Keywords:** VEMP, Chirp, Bone conducted, Tone Burst, Vestibular evoked myogenic potentials

## Abstract

**Purpose:**

Vestibular evoked myogenic potential (VEMP) is a test used to evaluate the function of otolith organs. In healthy individuals, air-conducted (AC) responses are obtained, whereas, in conductive hearing loss, the inability to transmit the signal well enough with AC stimuli has led to the need for bone-conducted (BC) stimuli. This study aimed to compare 500 Hz Chirp and Tone Burst stimuli in terms of latency and amplitude in BC cVEMP.

**Methods:**

This prospective observational case control study included 35 healthy participants (70 ears) between the age of 20–50. Participants underwent VEMP testing with BC 500 Hz Tone Burst stimulus and 500 Hz narrow band Chirp stimulus without changing the position of the bone conducted vibrator. The intensity of the stimuli was 50 dB nHL.

**Results:**

Response rate of 500 Hz TB Stimulus was 51.4% and 95.7% in Chirp stimulus. It was observed that significantly more responses were obtained with Chirp stimulus than TB stimulus (*p* < 0.001). In terms of p1 latency, n1 latency, both latencies were significantly shorter in Chirp stimulus (*p* < 0.001). p1n1 amplitude was found significantly larger in Chirp stimulus (*p* < 0.001).

**Conclusion:**

Compared to 500 Hz TB stimulus, 500 Hz Chirp stimulus results in a higher response rate, larger p1n1 amplitude, and shorter p1 and n1 latency. A higher response rate may provide a more accurate assessment of otolith organs, reducing false negatives due to signal transmission in patients.

## Introduction

The vestibular evoked myogenic potential (VEMP) is a vestibular test that assesses otolith function to help identify and diagnose vestibular disorders [1]. Cervical VEMPs (cVEMP) were first introduced by Colebatch in 1994 as a biphasic vestibular reflex derived from SCM with click stimuli. Calibrated headphones are used for air conducted (AC) stimulation and the responses obtained depend on short-term inhibition of the SCM muscle. It is known that cVEMP responses are obtained as a result of saccule stimulation [2].

The first study using bone conducted (BC) stimulation was performed by Halmagy in 1995 and it was shown that the sacculus could be stimulated using a tendon hammer tap [3]. In the following years, it has been demonstrated that this stimulation can also be achieved with B-71 bone-conductor [4]. The most important difference between the BC stimulus compared to the AC is that vestibular responses are obtained bilaterally, especially in a stimulus given at the midline of the skull. With the tendon hammer tap, good responses can be obtained from both the midline of the skull and the mastoid region, whereas with the B-71, better responses can be obtained from the mastoid region. [2]. In healthy individuals, both AC and BC responses are obtained, whereas, in conductive hearing loss, the inability to transmit the signal well enough with AC stimuli has led to the need for BC stimuli. At the same time, the lower intensity of the stimulus that needs to be delivered also provides a lower risk of cochlear damage [5].

Studies on BC cVEMP have mostly focused on tone burst stimulation and it has been stated that the most appropriate stimulus to be used to stimulate the saccule is 500 Hz tone burst stimulus. Changing the position or location of the B-71 vibrator modifies the response of otolith organs’ afferent neurons, which would be predicted to influence any myogenic response induced by neural stimulation [6]. This led us to search for a more powerful stimulus that would be less affected by position than the tone burst stimulus.

CE-Chirp stimulus, which has been used in recent years, has been reported to increase AC cVEMP amplitudes [7] and shorten latency [8, 9]. The CE-Chirp was created to promote temporal synchronization within the auditory system [10]. The term CE-Chirp is a licensed brand of Interacoustics, a Danish business that developed a family of short-duration acoustic stimuli for use in auditory evoked potential testing [1]. Narrowband (NB) Chirp can be used in four octave bands [8], but it is reported in the literature that the optimal stimulus for VEMP testing is 500 Hz NB Chirp [11].

There are very few publications on BC Chirp stimulus in the literature. In an article published by Çoban et al. BC Chirp was used in oVEMP test and a shortening in latencies and an increase in amplitudes were observed [12].

The aim of this study was to compare 500 Hz Chirp and Tone Burst stimuli in terms of latency and amplitude in BC cVEMP.

## Materials and methods

All procedures followed the Helsinki Declaration and were approved by University’s Ethics Committee (2023/2). 35 healthy participants (70 ears) were included (19 female, 16 male). Informed consent forms were obtained from all participants. Before starting the test, all participants underwent otological examination, audiometry, and tympanometry tests. Only participants with normal hearing and tympanometric results were included in the study.

The cVEMP test was conducted using Interacoustic Eclipse EP 15 (Interacoustics A/S, Middelfart, Denmark). The participants were told to preserve a seated posture. Participants’ skin was prepped with ethyl alcohol and scrub. Surface electromyographic (EMG) electrodes (Ambu Neuroline TM 720; Ambu, Ballerup, Denmark) were placed on the upper half of each SCM muscle, with reference electrodes on the supra-sternal notch and ground electrodes on the forehead. The electrodes’ impedance was kept below 5 k Ω. Tone bursts and NB chirp stimuli at 500 Hz were supplied in random order via a bone conductor vibrator (B71 model; Radioear Company, Minnesota/USA) placed on mastoid region.

**Fig. 1:**
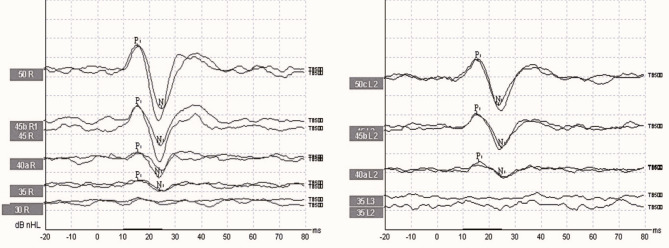
The waveform obtained with BC 500 Hz tone burst stimulus

To stimulate the 500 Hz tone burst, the stimulus strength was 50 dB nHL with a stimulus rate of 5.1/s. 200 stimuli were averaged with a rarefaction polarity. Rise/plateau/fall time of the stimulus was 2/1/2 ms. High pass filter was set to 10 Hz and low pass filter was set to 1000 Hz. The waveform obtained with BC 500 Hz tone burst stimulus is shown in Fig. 1.

**Fig. 2:**
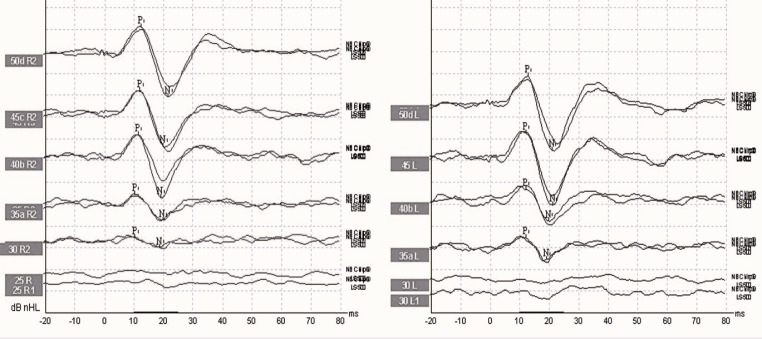
The waveform obtained with BC 500 Hz Chirp stimulus

The narrow band (NB) 500 Hz Chirp stimulus ranged from 360 to 720 Hz, was administered at 50 dB nHL intensity, and had a duration of 9 ms. The stimulus rate was set to 5.1/s, the analysis time to 55 ms, and polarity rarefaction. A total of 200 stimuli were averaged. The bone oscillator is not displaced during the transition from TB stimulus to Chirp stimulus. The waveform obtained with BC NB 500 Hz Chirp stimulus is shown in Fig. 2.

Potentials were approved when the waveform pattern and latencies were constant throughout at least two repetitions. Waves were tracked until the threshold was found. The waveforms’ P1 latency, N1 latency, and N1P1 amplitude were all assessed.

### Statistical analysis

The data was processed with SPSS 22 software (SPSS Inc., Chicago, IL, ABD). The distribution of the data was examined using the Shapiro-Wilk test. When the normal distribution was verified, the two groups were compared using the paired t-test. Alternatively, the groups were compared using the Wilcoxon test. Additionally, additional evidence regarding whether the data support the null hypothesis or the alternative hypothesis was provided using Bayesian statistics. Results of Bayesian statistics were given in posterior mean and 95% credible interval (CI). McNemar test was used to compare dependent categorical data. P-values < 0.05 were considered statistically significant.

## Results

Thirty-five patients (70 ears), including 19 females and 16 males, were enrolled in this study. The mean age of the patients was 27.29 ± 8.68. With 500 Hz tone burst stimulus, responses could be obtained from 36 ears (23 participants). With 500 Hz NB Chirp stimulus, responses could be obtained from 67 ears (35 participants).

Response rate of 500 Hz TB Stimulus was 51.4% (*n* = 36) and 95.7 (*n* = 67) in 500 Hz Chirp stimulus. When TB and Chirp stimuli were compared in terms of response rates, it was observed that significantly more responses were obtained with Chirp stimulus (McNemar Test, *p* < 0.001). Comparison of TB and Chirp stimuli in terms of response rates is shown in Table 1.

**Table 1:** Comparison of TB and chirp stimuli in terms of response rates

		500 Hz Chirp Stimulus				
		Absent (*n* = 3)	Present (*n* = 67)	Total (*n* = 70)	Test Stats*.	p
500 Hz Tone Burst Stimulus	Absent	2 (5.9%)	32 (94.1%)	34 (48.6%)	27.273	< 0.001
	Present	1 (2.8%)	35 (97.2%)	36 (51.4%)		

*McNemar test

When TB and Chirp stimuli were compared in terms of p1 latency, n1 latency, both latencies were significantly shorter in Chirp stimulus. p1n1 amplitude was found significantly larger in Chirp stimulus (*p* < 0.001). Interaural asymmetry was compared between TB and Chirp stimulus. There was no significant difference between these two stimuli (*p* = 0.477). Comparison of TB and Chirp stimuli in terms of response p1 latency, n1 latency, and p1n1 amplitude is shown in Table 2. When Bayes Factor for Related Samples T test was performed for p1, posterior mean was found 4.668, 95% CI was found 4.196–5.138. For n1 posterior mean was found 3.486, 95% CI was found 4.196–5.138. For Amplitude posterior mean was found − 29.588, 95% CI was found between − 38.538 and − 20.638.

**Table 2:** Comparison of TB and chirp stimuli in terms of p1, n1 latencies, and p1n1 amplitudes

	500 Hz TB		500 Hz Chirp			
	Mean ± SD	Median (min-max)	Mean ± SD	Median (min-max)	Test Stats	p
p1 (ms)	16.17 ± 24.02	15.67 (13-20.67)	11.64 ± 20.09	11.33 (9–17)	-5.446^a^	< 0.001
n1 (ms)	24.02 ± 38.1	24 (20-28.33)	20.09 ± 0	20 (15.33–25.67)	11.373^b^	< 0.001
Amplitude (µV)	38.1 ± 0.2	27.72 (11.13–162.5)	54.67 ± 34.98	42.78 (13.82–170)	-4.996^a^	< 0.001

^a^: Wilcoxon signed-rank test, ^b^: Paired samples T test, SD: Standart deviation, min: minimum, max: maximum, ms: millisecond, µV: microvolt

No significant difference was found between TB and Chirp stimulus in terms of interaural asymmetry (Z=-0.711, *p* = 0.477).

## Discussion

When patients have ACS VEMP testing and there is no response, it is unclear if this is due to the stimulus procedure, middle ear pathology, or a real vestibulopathy [13]. In various studies available in the literature, it has been shown that the obtainability of VEMP responses decreases with AC stimuli in conductive hearing loss [13, 14]. For this reason, studies have been directed towards the use of BC stimuli in conductive hearing loss.

There are articles in the literature comparing the results of tendon hammer tap, Radioear B-71 and mini-shaker [13]. Iwasaki et al. (2008) compared mini-shaker and Radioear B-71 in BC oVEMP and reported that B-71 did not provide sufficient stimulation [15]. However, since different transducers will not be available in every clinic, we aimed to obtain bone tract responses with a different stimulus.

Curthoys et al. (2010) reported that the position of the bone oscillator on the skull may also influence the results [6]. Therefore, in our study, we switched between stimuli without changing the location of the oscillator. The fact that we were able to get a response with Chirp stimulus in participants who could not respond with TB stimulus made us conclude that Chirp stimulus is a better stimulus than TB stimulus in BC cVEMP.

There are several studies comparing 500 Hz TB and 500 Hz Chirp stimuli in AC cVEMP and oVEMP. Murofushi et al. (2020) observed a shortening in p1 latency and a decrease in p1n1 amplitude with chirp stimulus. They found no significant difference between the two stimuli in terms of response rate [16]. Ocal et al.(2021) reported shorter p1 and n1 latencies with a larger p1n1 amplitude [17]. We obtained similar results with BC Chirp in accordance with the findings of Ocal et al. Aydın et al. (2021) also compared TB and Chirp stimuli in both oVEMP and cVEMP and obtained shorter p1 and n1 latencies, higher p1n1 amplitude, and higher response rate with Chirp stimulus [18]. However, as far as we know, there is no publication comparing BC 500 Hz TB and Chirp stimuli.

Çoban et al. (2021) compared 500 Hz TB and 500 Hz NB Chirp stimulus in BC oVEMP test and observed a shortening in n1 and p1 latencies and an increase in n1p1 amplitude with Chirp stimulus [12]. Similarly, we obtained shorter p1 and n1 latencies and higher p1n1 amplitude with Chirp stimulus. The results can be explained using numerous facts. One of the basis is that the tone burst stimulus has a substantially longer rise/fall time, which reduces its potency. Unlike tone bursts, chirp stimuli have no rise/fall time. The latency of reactions in BC oVEMP is made up of three components: bone conduction through the skull, transit time inside the utricle, and neural response. Çoban et al. stated that Chirp stimulus has a better stability than TB stimulus. They also suggested that the long latencies and low amplitudes obtained with TB stimuli may be due to frequency scattering [12]. The same may be possible in our study.

The main limitation of this study is that BC stimulation not only stimulated the saccule but also the utricle, thus obtaining a whole otolith organ response.

Compared to 500 Hz TB stimulus, 500 Hz Chirp stimulus results in higher response rate, larger p1n1 amplitude and shorter p1 and n1 latency. A larger wave amplitude may increase the recognizability of the waves, facilitating threshold determination. A higher response rate may provide more accurate assessment of otolith organs, reducing false negatives due to signal transmission in patients.
